# Identification of Potentially Functional Circular RNA/Long Noncoding RNA-MicroRNA-mRNA Regulatory Networks Associated with Vascular Injury in Type 2 Diabetes Mellitus by Integrated Microarray Analysis

**DOI:** 10.1155/2023/3720602

**Published:** 2023-03-10

**Authors:** Yi Leng, Ming-zhu Wang, Kang-ling Xie, Ying Cai

**Affiliations:** ^1^Department of Rehabilitation, Xiangya Hospital, Central South University, Changsha, Hunan 410008, China; ^2^Department of Orthopedics, Xiangya Hospital, Central South University, Changsha, Hunan 410008, China; ^3^National Clinical Research Center for Geriatric Disorders, Xiangya Hospital, Central South University, Changsha, Hunan 410008, China

## Abstract

This research is aimed at figuring out the potential circular RNA (circRNA)/long noncoding RNA- (lncRNA-) microRNA- (miRNA-) mRNA regulatory networks associated with a vascular injury in type 2 diabetes mellitus (T2DM). Differentially expressed genes (DEGs) screened in T2DM-related expression datasets were intersected with genes associated with vascular injury in T2DM to obtain candidate DEGs, followed by the construction of an interaction network of DEGs. The upstream miRNAs of candidate genes were predicted by mirDIP, miRWalk, and DIANA TOOLS databases, and the upstream lncRNAs/circRNAs of miRNAs by DIANA-LncBase/circBank database, followed by the construction of circRNA/lncRNA-miRNA-mRNA regulatory networks. Peripheral blood was attained from T2DM patients with macroangiopathy for clinical validation of expression and correlation of key factors. Differential analysis screened 37 candidate DEGs correlated with vascular injury in T2DM. Besides, MAPK3 was a core gene associated with vascular injury in T2DM. Among the predicted upstream miRNAs of MAPK3, miR-4270, miR-92a-2-5p, miR-423-5p, and miR-613 ranked at the top according to binding scores. The upstream lncRNAs and circRNAs of the 4 miRNAs were further predicted, obtaining 11 candidate lncRNAs and 3 candidate circRNAs. Moreover, KCNQ1OT1, circ_0020316, and MAPK3 were upregulated, but miR-92a-2-5p was downregulated in the peripheral blood of T2DM patients with macroangiopathy. Mechanistically, KCNQ1OT1 and circ_0020316 bound to miR-92a-2-5p that inversely targeted MAPK3. Collectively, KCNQ1OT1/circ_0020316-miR-92a-2-5p-MAPK3 coexpression regulatory networks might promote vascular injury in T2DM.

## 1. Introduction

Type 2 diabetes mellitus (T2DM) accounts for more than 90% of patients with diabetes and is featured with relative insulin deficiency resulting from pancreatic *β*-cell dysfunction and insulin resistance in target organs [1]. T2DM is a multifactorial metabolic disease that is attributable to the interplay between multiple environmental and genetic predispositions [2]. T2DM results in microvascular and macrovascular complications, which leads to severe psychological and physical distress to both patients and careers and imposes a massive burden on the health care system [3]. Besides, T2DM can adversely impact the microvasculature in various organs, and therefore, T2DM may develop microvascular injury/dysfunction as a chronic outcome [4]. Therefore, the vascular injury may assume a role in T2DM. This background calls for the necessary exploration of the molecular mechanism behind vascular injury in T2DM.

Circular RNA (circRNA) is a novel kind of endogenous noncoding RNA with tissue-specific and cell-specific expression patterns. It has been demonstrated to be involved in multiple diseases like neurological disorders, cancer, diabetes mellitus, and cardiovascular diseases [5]. It was previously reported that hsa_circ_0054633 could be utilized as a diagnostic biomarker of T2DM [6]. Another report elucidated that circANKRD36 was linked to inflammation in T2DM patients [7]. As transcripts of more than 200 nucleotides that are not translated into proteins, long noncoding RNAs (lncRNAs) have been documented to participate in the physiology of tissues and organs and disease processes by orchestrating numerous cell processes, including cell division, differentiation, survival, and senescence [8, 9]. Recently, lncRNA MALAT1 was found to reduce insulin resistance in T2DM [10]. Additionally, lncRNA MEG3 could assume a crucial role in T2DM-induced vascular disease [11]. Therefore, these findings illustrated that circRNAs and lncRNAs might function as novel potential biomarkers for T2DM.

Recently, great attention has been paid to the competing endogenous RNA (ceRNA) regulatory network that lncRNAs/circRNAs act as a sponge for microRNAs (miRNAs/miRs) to indirectly upregulate miRNA downstream target genes, thus modulating disease progression [12, 13]. For instance, mmu_circ_0000250 enhanced wound healing in diabetes by upregulating SIRT1 through binding to miR-128-3p [14]. In addition, LINC-P21 could repress insulin secretion and proliferation of pancreatic *β*-cells in T2DM through binding to miR-766-3p to upregulate NR3C2 [15].

With the rapid development of sequencing and large sample analysis, bioinformatics analysis has been widely utilized in biological research and therapeutic progress [16]. It has been widely used to analyze public databases to construct a more comprehensive lncRNA/circRNA-miRNA-mRNA regulatory network and mine more accurate prognostic markers [17]. Moreover, there exist few researches to predict the prognostic lncRNA-miRNA-mRNA [18] and circRNA/lncRNA-miRNA-mRNA [19] networks in T2DM. Gene Expression Omnibus (GEO) database can provide circRNA, miRNA, and mRNA of numerous diseases, which can be applied in data mining and biological discovery [20].

In our study, we aimed to figure out the potential circRNA/lncRNA-miRNA-mRNA regulatory networks associated with vascular injury in T2DM. Our study provided a theoretical basis for further understanding the pathogenesis of T2DM vascular injury and searching for potential diagnostic and therapeutic targets.

## 2. Materials and Methods

### 2.1. Ethics Statement

Written informed consents were acquired from all participants prior to enrollment. Study protocols were ratified by the Ethics Committees of our hospital and strictly adhered to the *Declaration of Helsinki*.

### 2.2. Acquisition and Analysis of Data in Expression Datasets from GEO Database

The T2DM-related expression datasets GSE15653 and GSE21340 were attained from GEO database. The GSE15653 dataset encompasses 9 liver biopsy tissue samples from T2DM obese patients and 5 obese without T2DM samples. The GSE21340 dataset consists of 15 plasma samples from T2DM patients and 5 normal control samples.

Differentially expressed genes (DEGs) in these 2 datasets were identified using the R language “limma” package with a threshold of *p* value < 0.05. Volcano plots were drawn using the R language “ggplot2” package. Meanwhile, the correlation analysis of mRNA expression of candidate genes was performed using the R language “corrplot” package.

### 2.3. Retrieval of Disease-Related Database

T2DM-related target genes were researched through databases of GeneCards (relevance score ≥ 20) and Comparative Toxicogenomics Database (CTD; inference score ≥ 30).

### 2.4. Functional Enrichment Analysis of Candidate Genes

Venn analysis of the result of microarray analysis and databases of GeneCards and CTD was conducted using the draw Venn diagram tool to identify candidate genes. Gene Ontology (GO) and Kyoto Encyclopedia at Genes and Genomes (KEGG) enrichment analyses were performed on candidate genes using the R language “ClusterProfiler” package to analyze the cellular functions and signaling pathways that were mainly impacted by potential targets and key targets. *p* < 0.05 was considered statistically significant.

### 2.5. Construction of Protein Interaction Network of Candidate Genes

The interaction network of target genes was obtained through STRING with species condition limited to “Homo sapiens” to construct the regulatory relationship network. Minimum required interaction score was set as 0.4. Active interaction sources, including text mining, experiments, databases, coexpression, neighborhood, gene fusion, and cooccurrence, were used to construct a network of regulatory relationships. Then, the PPI enrichment value less than 1.0*e*-16 in enrichment network was selected to import into Cytoscape (v3.8.2) software. The network relationship plots were subjected to result analysis and ranking. The degree value and combine score value were indicated by colors, and candidate genes were ranked based on the degree value.

### 2.6. Prediction of Upstream miRNA-lncRNA/circRNA of Candidate Genes

The upstream miRNAs of candidate target genes were predicted using mirDIP, miRWALK, and DIANA TOOLS databases, which were intersected by the draw Venn diagram tool. The upstream lncRNAs of miRNAs were predicted by DIANA-LncBase database, and the upstream circRNAs of miRNAs were predicted by circBank database. Afterwards, the lncRNA/circRNA-miRNA-mRNA coexpression regulatory network was constructed, which was visualized using Cytoscape (v3.8.2).

### 2.7. Clinic Sample Collection

A total of 50 T2DM patients with macroangiopathy (36 males and 14 females; mean age: 54.5 ± 7.1 years) treated in our hospital from January 2020 to January 2021 were enrolled as the case group. In addition, 30 normal healthy individuals (15 males and 15 females; mean age: 55.2 ± 4.9 years) who underwent physical examination during the same period were enrolled as the control group. Fasting peripheral blood (5 mL) was harvested from all participants during admission or the early morning of the physical examination day. All patients were diagnosed for the first time and had no history of long-term medication, cancer, or chronic diseases.

### 2.8. Reverse Transcription Quantitative Polymerase Chain Reaction (RT-qPCR)

Total RNA was extracted from peripheral blood of healthy controls and T2DM patients with macroangiopathy using a TRIzol Kit (Invitrogen, Carlsbad, California, USA) and reversely transcribed to cDNA using as per the manuals of TaqMan MicroRNA Assays Reverse Transcription Primer (4427975, Applied Biosystems, Carlsbad, CA, USA). lncRNA, circRNA, and mRNA expressions were normalized by glyceraldehyde-3-phosphate dehydrogenase (GAPDH), and miRNA expression was normalized by U6. Relative differences in target gene expression were calculated using a 2^-*ΔΔ*CT^. Primers are manifested in Supplementary Table 1 (primer design using NCBI's primer design function).

### 2.9. Human Vascular Smooth Muscle Cell (HVSMC) Culture

HVSMC (bio-73393, Biobw, Beijing, China) were cultured with Ham's F-12K medium (PM150910, Biobw) encompassing 0.05 mg/mL vit. C+0.01 mg/mL insulin+0.01 mg/mL transferrin+10 ng/mL sodium selenite+0.03 mg/mL endothelial cell growth supplement+10% fetal bovine serum+10 mM 4-(2-hydroxyethyl)-1-piperazineëthanesulfonic acid+10 mM TES+1% P/S in a 37°C with 5% CO_2_ and saturated humidity. The cells were passaged by trypsinization when they reached more than 90% confluence, and the cells in the exponential growth phase were used for follow-up experiments.

### 2.10. Dual Luciferase Gene Reporter Assay

The 3′ untranslated region (UTR) of the mitogen-activated protein kinase 3 (MAPK3) gene was clonally amplified, and the PCR product was cloned into the multicloning site at downstream of the luciferase gene of pmirGLO (E1330, Promega, Madison, WI, USA) luciferase gene, named as pMAPK3 wild type (WT). The pMAPK3 mutant type (MUT) vector was constructed by site-directed mutagenesis of the binding sites for bioinformatically predicted miR-92a-2-5p to target genes. With pRL-TK vector expressing Renilla luciferase (E2241, Promega) as an internal reference, miR-92a-2-5p mimic and negative control were cotransfected with luciferase reporter vector, respectively, into HVSMCs, and luciferase activity was detected according to the method provided by Promega.

### 2.11. Statistical Analysis

Statistical analyses were performed using SPSS21.0 software (IBM Corp. Armonk, NY, USA). Measurement data were described as mean ± standard deviation. Data between the two groups were compared using an unpaired *t*-test. Correlation analysis was performed by the Pearson method. A value of *p* < 0.05 was regarded as statistically significant.

## 3. Results

### 3.1. There Were 37 Candidate Genes Associated with Vascular Injury in T2DM

**Figure 1:**
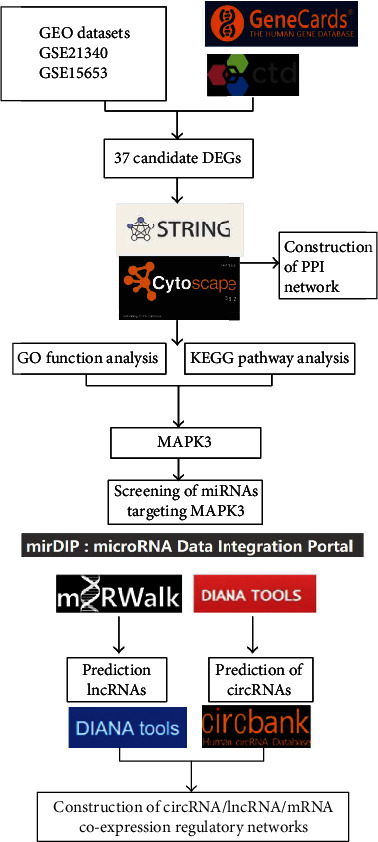
Flow chart of bioinformatics analysis.

We screened out the key factors associated with vascular injury in T2DM by GEO database. Then, circRNA/lncRNA-miRNA-mRNA coexpression networks were constructed, followed by further exploration of the biological functions of the circRNA/lncRNA-miRNA-mRNA coexpression networks in the occurrence of vascular injury in T2DM. The flow of bioinformatics screening of key genes involved in the development of vascular injury in T2DM is detailed in Figure 1.

**Figure 2:**
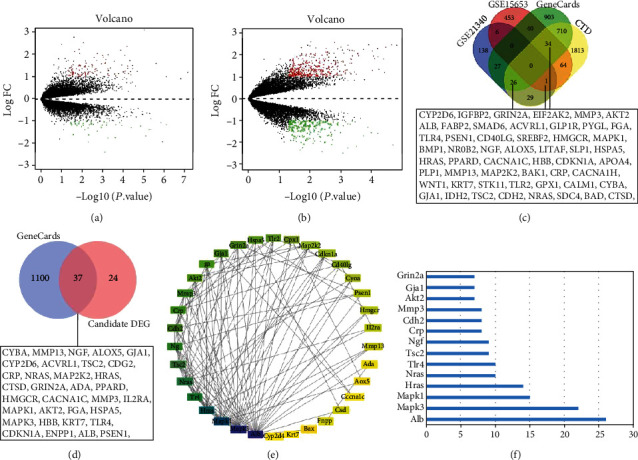
Screening of genes associated with vascular injury in T2DM using datasets in GEO database. (a) Differentially expressed mRNAs in T2DM samples and normal samples from GSE21340 and GSE15653. (b) Differentially expressed mRNAs in T2DM samples and normal samples from GSE15653. Green dots indicated downregulated genes, red dots indicated upregulated genes, and gray dots indicated genes with no significant difference. (c) Venn diagram of DEGs in GSE21340 and GSE15653 datasets and results from GeneCards and CTD databases. (d) Venn analysis of screening genes in panel (c) and vascular injury-related genes in GeneCards database. (e) Interaction network diagram of the 37 candidate DEGs (nodes represented proteins and edges represented protein-protein associations). (f) The degree value of the candidate DEGs.

First, 227 DEGs were obtained from the T2DM-associated dataset GSE21340, of which 110 DEGs were upregulated and 117 DEGs were downregulated (Figure 2(a)). Then, 598 DEGs were identified in the T2DM-associated GSE15653 dataset, among which 373 DEGs were upregulated and 225 were downregulated (Figure 2(b)). The T2DM-associated genes were retrieved through GeneCards database, and 1470 genes were filtered using relevance score ≥ 20, whereas 2677 genes were filtered from the CTD database using inference score ≥ 30. The DEGs from GSE21340 and GSE15653 were intersected with the results of GeneCards and CTD databases, which filtered out 61 candidate DEGs (Figure 2(c)).

Further vascular injury-related genes were retrieved from GeneCards database, and 1137 genes were filtered by relevance score ≥ 5, which was then intersected with the above 61 candidate DEGs. A total of 37 candidate DEGs related to vascular injury were harvested (Figure 2(d)). Subsequently, the 37 candidate DEGs were imported into the STRING database with the corresponding filtering conditions to obtain protein interaction relationships which were then imported into Cytoscape software to construct the protein-protein interaction (PPI) network. There existed 37 nodes and 116 edges in the PPI network relationship graph (PPI enrichment *p* value < 1.0*e*-16): a larger shape indicated a larger degree value, and the greater the degree value and combine score value corresponded to the color from yellow to purple (Figure 2(e)). In the network, the top 5 genes by degree value were albumin (Alb), MAPK3, MAPK1, Hras, and Nras, respectively (Figure 2(f)).

### 3.2. MAPK3 Might Be a Core Gene in the Development of Vascular Injury in T2DM

**Figure 3:**
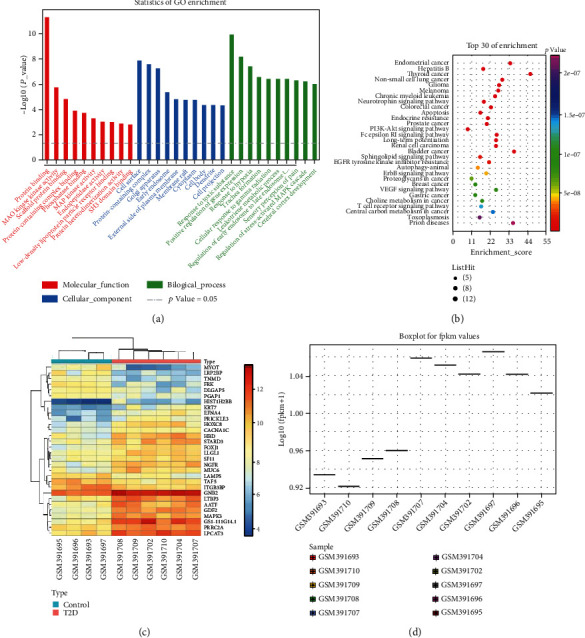
Secondary screening of core genes involved in the development of vascular damage in T2DM. (a) GO functional analysis of candidate DEGs at the BP, CC, and MF levels. (b) KEGG pathway enrichment analysis of candidate DEGs. Dot size indicated the number of selected genes, and color represented the *p* value for enrichment analysis. (c) Heat maps of candidate DEG expression matrix in different samples; the warmer the color of the block, the higher the expression. (d) MAPK3 expression in T2DM in GSE15653.

These 37 candidate DEGs further underwent GO and KEGG enrichment analyses. The GO functional analysis results indicated that in terms of biological processes (BP), DEGs were mainly enriched in positive regulation of gene expression, response to hypoxia, regulation of stress-activated MAPK cascade, and inflammatory response; in terms of cellular component (CC), DEGs were mainly enriched in protein binding, MAP kinase activity, scaffold protein binding, protein-containing complex binding, and MAP kinase activity; in terms of molecular function (MF), DEGs were mainly enriched in protein-containing complex, Golgi apparatus, early endosome, and external side of plasma membrane (Figure 3(a)). As reflected by KEGG pathway analysis results, DEGs were mainly enriched in vascular endothelial growth factor signaling pathway, apoptosis, phosphoinositide 3-kinase-Akt signaling pathway, MAPK signaling pathway, and ErbB signaling pathway (Figure 3(b)). These results suggested that DEGs mainly assumed a role in MAPK cascade, inflammatory response, and stress activation and were enriched in structures such as mitochondria, extracellular space, and cytoplasm. In addition, the MF of DEGs mainly manipulated cell-related enzyme activities and protein binding.

In the progression of T2DM, vascular system disease is one of the critical complications, and the mechanism of vascular injury is associated with the pathogenesis of insulin resistance, diabetic nephropathy, and peripheral arterial disease [21]. When T2DM patients experience hypoglycemia, it promotes the release of inflammatory factors, increases the aggregation of platelets, alters hemodynamics, damages vascular endothelial cells, and enhances the incidence of cardiovascular system disease [22]. It has been documented that inhibition of the MAPK3 signaling pathway decreases VSMC proliferation and migration to alleviate vascular neointimal hyperplasia induced by vascular injury in rats [23]. In addition, GSE15653 analysis manifested that MAPK3 was remarkably highly expressed in T2DM (Figures 3(c) and 3(d)). Therefore, we speculated that MAPK3 might be the core gene of T2DM-induced vascular injury.

### 3.3. Screening Upstream miRNAs and circRNAs/lncRNAs of MAPK3 and Coexpression Network Construction

**Figure 4:**
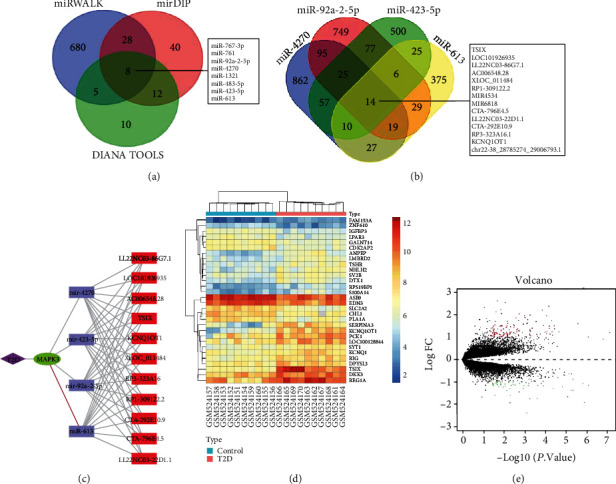
Prediction of upstream miRNAs and lncRNAs modulating MAPK3 by online database. (a) Venn analysis of the upstream miRNAs of MAPK3 predicted by mirDIP, miRWalk, and DIANA TOOLS databases. (b) Venn analysis of the upstream lncRNAs of miR-4270, miR-92a-2-5p, miR-423-5p, and miR-613 predicted by DIANA-LncBase database. (c) Construction of lncRNA-miRNA-MAPK3 coexpression regulatory network by Cytoscape. (d) Heat map of GSE20966 analysis. (e) Volcano plots of GSE20966.

In the mechanism of vascular injury development in T2DM, miRNAs, lncRNAs, and circRNAs are involved in mediating vascular endothelial cell injury and repair capacity [24, 25]. The upstream miRNAs of MAPK3 were further predicted by 3 common online databases (mirDIP, miRWALK, and DIANA TOOLS) with the species as human. In total, 88 miRNAs were predicted by mirDIP database, 721 miRNAs were predicted by miRWALK database, and 35 miRNAs were predicted by DIANA TOOLS database (score > 0.5), the intersection of which was obtained to screen out 8 miRNAs (Figure 4(a)). The scores of target binding to MAPK3 for the 8 miRNAs screened are depicted in Supplementary Table 2, among which miR-4270, miR-92a-2-5p, miR-423-5p, and miR-613 ranked at the top and all participated in T2DM or vascular injury [26–29].

The upstream lncRNAs of miR-4270, miR-92a-2-5p, miR-423-5p, and miR-613 were further predicted through DIANA-LncBase database, obtaining 1109, 1014, 714, and 505 lncRNAs, respectively. Moreover, 14 intersected lncRNAs were screened by Venn analysis (Figure 4(b)). The lncRNA data that could not be utilized for subsequent experiments were excluded by querying the NCBI website, which obtained 11 lncRNAs, and the specific binding relationships are displayed in Supplementary Table 3. The network regulation diagram was drawn by Cytoscape software, and the lncRNA-miRNA-MAPK3 coexpression regulatory network was constructed (Figure 4(c)). Furthermore, TSIX, KCNQ1OT1, and LOC101926935 were upregulated in T2DM of GSE20966 (Figures 4(d) and 4(e)).

**Figure 5:**
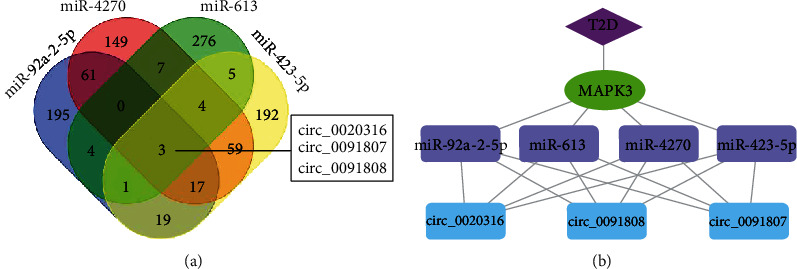
Prediction of upstream circRNAs orchestrating MAPK3 by online database. (a) Venn analysis of the upstream circRNAs of miR-4270, miR-92a-2-5p, miR-423-5p, and miR-613 predicted by circBank database. (b) Construction of circRNA-miRNA-MAPK3 coexpression regulatory network by Cytoscape.

Further, the circBank database was adopted to predict the upstream circRNAs of miR-4270, miR-92a-2-5p, miR-423-5p, and miR-613, and the top 300 targeted circRNAs were, respectively, subjected to Venn analysis to yield three candidate circRNAs (circ_0020316, circ_0091807, and circ_0091808) in the intersection (Figure 5(a)). The specific binding relationships are manifested in Supplementary Table 4. Then, Cytoscape software was applied to draw a network regulatory relationship diagram and construct a circRNA-miRNA-MAPK3 regulatory network (Figure 5(b)).

### 3.4. KCNQ1OT1, circ_0020316, and MAPK3 Expressions Were High, but miR-92a-2-5p Expression Was Low in Peripheral Blood of T2DM Patients with Macroangiopathy

**Figure 6:**
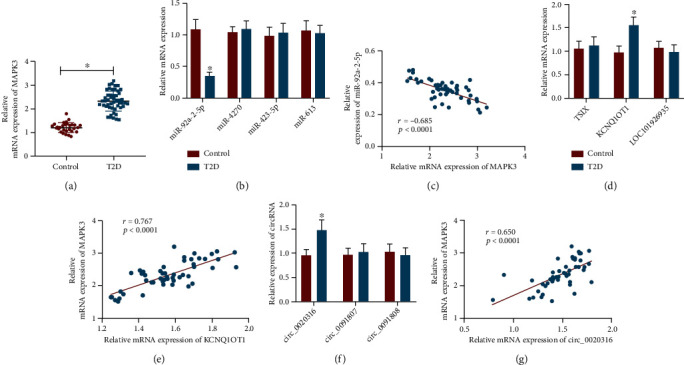
KCNQ1OT1, circ_0020316, and MAPK3 are upregulated, but miR-92a-2-5p is downregulated in peripheral blood of T2DM patients with macroangiopathy. (a) RT-qPCR detection of MAPK3 mRNA expression in peripheral blood of 50 T2DM patients with macroangiopathy and 30 normal healthy controls. (b) miR-4270, miR-92a-2-5p, miR-423-5p, and miR-613 expressions determined in peripheral blood of 50 T2DM patients with macroangiopathy and 30 normal healthy controls by RT-qPCR. (c) Correlation between miR-92a-2-5p and MAPK3 expressions assessed by Pearson's methods. (d) TSIX, KCNQ1OT1, and LOC101926935 expressions in peripheral blood of 50 T2DM patients with macroangiopathy and 30 normal healthy controls measured by RT-qPCR. (e) Correlation between KCNQ1OT1 and MAPK3 expressions assessed by Pearson's methods. (f) circ_0020316, circ_0091807, and circ_0091808 expressions in peripheral blood of 50 T2DM patients with macroangiopathy and 30 normal healthy controls measured by RT-qPCR. (g) Correlation between circ_0020316 and MAPK3 expressions evaluated by Pearson's methods. ^∗^*p* < 0.05, compared with normal healthy controls.

To identify the mechanism of MAPK3 and its upstream miRNAs, lncRNAs, and circRNAs in vascular injury of T2DM, peripheral blood samples were harvested from 50 T2DM patients with macroangiopathy and 30 normal healthy controls. RT-qPCR revealed that compared with healthy control, MAPK3 mRNA expression was obviously augmented in the peripheral blood of T2DM patients (Figure 6(a)), whereas miR-92a-2-5p expression was potently diminished (Figure 6(b)) and negatively correlated with MAPK3 expression (Figure 6(c)). In addition, KCNQ1OT1 and circ_0020316 expressions was noticeably enhanced in the peripheral blood of T2DM patients compared with healthy controls and exhibited positive correlations with MAPK3 expression (Figures 6(d)–6(g)). Conclusively, KCNQ1OT1/circ_0020316-miR-92a-2-5p-MAPK3 coexpression regulatory networks may be a key molecular pathway involved in the development of vascular injury in T2DM.

### 3.5. KCNQ1OT1 and circ_0020316 Bound to miR-92a-2-5p to Upregulate MAPK3

**Figure 7:**
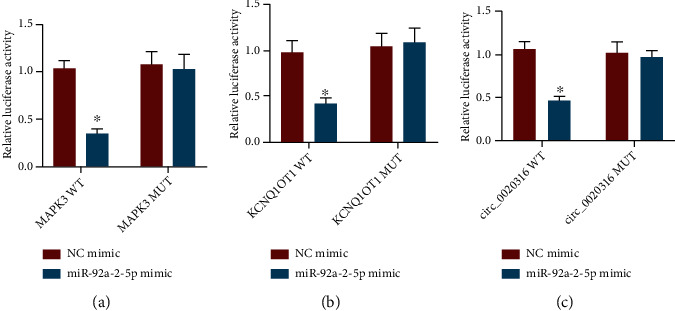
KCNQ1OT1 and circ_0020316 manipulate MAPK3 by binding to miR-92a-2-5p. (a) The targeting relationship between MAPK3 and miR-92-a-2-5p evaluated by dual luciferase gene reporter assay. (b) The binding relationship between KCNQ1OT1 and miR-92-a-2-5p evaluated by dual luciferase gene reporter assay. (c) The binding relationship between circ_0020316 and miR-92-a-2-5p evaluated by dual luciferase gene reporter assay. ^∗^*p* < 0.05, NC mimic treatment.

Dual luciferase gene reporter assay results manifested that miR-92a-2-5p mimic reduced luciferase activity of MAPK3-WT but did not impact that of MAPK3-MUT in HEK293 cells (Figure 7(a)), indicating that MAPK3 was negatively targeted by miR-92a-2-5p.

Moreover, the luciferase activities of KCNQ1OT1-WT and circ_0020316-WT were diminished by miR-92a-2-5p mimic, while there was no obvious difference in luciferase activities of KCNQ1OT1-MUT and circ_0020316-MUT (Figures 7(b) and 7(c)). Therefore, both KCNQ1OT1 and circ_0020316 bound to miR-92a-2-5p.

## 4. Discussion

T2DM is a prevalent disease resulting in major neurologic, renal, and vascular complications, and it is of significance to prevent and treat T2DM and its complications [30]. The clinical management of T2DM is through a healthy diet and lifestyle combined with glucose-lowering agents aimed at preventing or delaying the acute symptoms of hyperglycemia and complications of the disease [31]. In spite of the therapeutic benefits of glucose-lowering agents for the treatment of T2DM, most of the drugs can contribute to some undesirable side effects [32]. Therefore, it is imperative to deepen the understanding of biomarkers for the diagnosis and treatment of T2DM. Considering this, this research was conducted through bioinformatics analysis and experiments on peripheral blood from T2DM patients and revealed that KCNQ1OT1/circ_0020316-miR-92a-2-5p-MAPK3 regulatory networks might promote vascular injury in T2DM.

Initially, we screened out 37 candidate DEGs associated with vascular damage in T2DM through differential analysis of T2DM-associated datasets GSE15653 and GSE21340, among which Alb, MAPK3, MAPK1, Hras, and Nras were the top five genes ranked by the degree value in the PPI network. A prior research elucidated that dynamic change of serum Alb level was correlated with T2DM risk [33]. Also, it was noted in another research that MAPK3 and MAPK1 were involved in heart failure caused by diabetes [34]. Hras has been demonstrated to accelerate glucose-induced apoptosis of retinal capillary cells in diabetes [35]. Besides, diabetes could elevate Nras expression during rat oral oncogenesis [36]. Moreover, combined with the results of PPI network analysis, GO analysis, and KEGG analysis, the present research indicated that MAPK3 might be a core gene associated with vascular injury in T2DM. Additionally, RT-qPCR revealed that MAPK3 was upregulated in peripheral blood from T2DM patients. Consistently, it was predicted by the research of Du and Uversky that MAPK3 was a moderately disordered protein in T2DM [37]. Moreover, integrated microarray analysis in the research of Li et al. manifested predicted MAPK3 as the hub gene in circMYO9B/circGRAMD1B/circTHAP4/circTMC7-miRNA-mRNA regulatory network in T2DM [38]. Of note, another report predicted that MAPK3 might be involved in antidiabetic and antihyperlipidemic effects of Gegen in T2DM [39]. Also, it was previously reported that MAPK3A was differentially expressed in atherosclerosis, a common chronic vascular inflammatory disease, which suggested the potential role of MAPK3 in vascular injury [40].

It has been generally accepted that lncRNAs/circRNAs may bind to miRNAs through their miRNA response elements, thus orchestrating the expression of target genes of miRNAs [41]. Based on this, we further predicted the circRNA/lncRNA-miRNA-MAPK3 network involved in vascular injury in T2DM, which was further screened by clinic sample experiments and dual luciferase gene reporter assay. Our data documented that KCNQ1OT1/circ_0020316-miR-92a-2-5p-MAPK3 regulatory networks were involved in vascular injury in T2DM. Corroborating findings were reported in a prior work that miR-92a was poorly expressed in animal models of diabetes [42]. Moreover, mmu-miR-92a-3p upregulation was involved in the rescue of diabetes-impaired angiogenesis by reconstituted high-density lipoproteins, which suggested its implications for the treatment of diabetes-related vascular complications [43]. Of note, the involvement of KCNQ1OT1 has been displayed in T2DM susceptibility [44]. Yang et al. observed KCNQ1OT1 upregulation in patients with diabetes, high glucose-induced cardiomyocytes, and diabetic mouse cardiac tissue, and silencing KCNQ1OT1 inhibits diabetic cardiomyopathy [45]. In addition, a prior study uncovered that KCNQ1OT1 was overexpressed in diabetic nephropathy and that its silencing depressed proliferation and fibrosis and elevated apoptosis in diabetic nephropathy cells [46]. circRNAs have been implicated in vascular diseases, including vascular dysfunction, diabetes mellitus-related retinal vascular dysfunction, and hepatic vascular invasion in T2DM [47]. For instance, cZNF532 upregulation alleviated human diabetes-induced retinal pericyte degeneration and vascular dysfunction [48]. circHIPK3 silencing contributed to the enhancement of retinal endothelial cell proliferation, migration, and tube formation, thus depressing retinal vascular dysfunction in diabetes [49]. Of note, our research predicted the involvement of a novel circRNA, circ_0020316, in the circRNA-miRNA-MAPK3 network in vascular injury in T2DM. Furthermore, clinic sample detection showed that circ_0020316 and KCNQ1OT1 expressions were high in the peripheral bloods of T2DM patients, and dual luciferase gene reporter assay documented that circ_0020316 and KCNQ1OT1 bound to miR-92a-2-5p.

## 5. Conclusion

**Figure 8:**
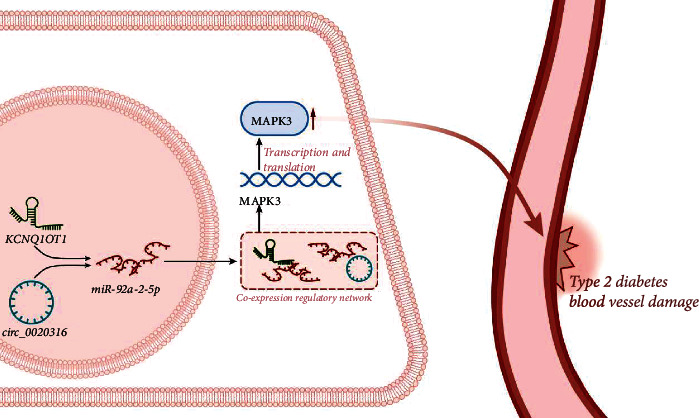
Mechanism of KCNQ1OT1/circ_0020316-miR-92a-2-5p-MAPK3 coexpression regulatory networks in vascular injury in T2DM. KCNQ1OT1/circ_0020316-miR-92a-2-5p-MAPK3 coexpression regulatory networks may contribute to the development of vascular injury in T2DM by elevating MAPK3 expression.

Conclusively, our data revealed the involvement of KCNQ1OT1/circ_0020316-miR-92a-2-5p-MAPK3 regulatory networks in T2DM-induced vascular injury (Figure 8). Our results provide new insight for mechanistic investigations and may offer potential therapeutic targets for T2DM. Future studies are needed to better understand the role of these two regulatory networks in vascular injury in T2DM.

## Data Availability

The datasets generated and/or analyzed during the current study are available in the manuscript and supplementary materials.

## Supplementary Materials

Supplementary Materials Supplementary Table 1: primers used for RT-qPCR. Supplementary Table 2: the target binding scores of miRNAs to MAPK3. Supplementary Table 3: the binding of lncRNAs to miRNAs. Supplementary Table 4: the binding of circRNAs to miRNAs.
