# Supplementary material for: Identification of Potentially Functional Circular RNA/Long Noncoding RNA-MicroRNA-mRNA Regulatory Networks Associated with Vascular Injury in Type 2 Diabetes Mellitus by Integrated Microarray Analysis
Source: J Diabetes Res. 2023 Mar 10;2023:3720602. doi: 10.1155/2023/3720602 (PMC10023230; doi:10.1155/2023/3720602)
Supplement: Supplementary Materials — Supplementary Table 1: primers used for RT-qPCR. Supplementary Table 2: the target binding scores of miRNAs to MAPK3. Supplementary Table 3: the binding of lncRNAs to miRNAs. Supplementary Table 4: the binding of circRNAs to miRNAs. [file 3720602.f1.docx]

**Supplementary Table 1** Primers used for RT-qPCR

| Names | Primer sequence (5'-3') |
| --- | --- |
| MAPK3 | Forward: CATGGAGCTGGATGACCTACC |
|  | Reserve: TATCAGGGTGTCCATGTGTGG |
| miR-4270 | Forward: CGCGTCAGGGAGTCAGGG |
|  | Reserve: AGTGCAGGGTCCGAGGTATT |
| miR-92a-2-5p | Forward: CGGGGTGGGGATTTGTTG |
|  | Reserve: AGTGCAGGGTCCGAGGTATT |
| miR-423-5p | Forward: GTGAGGGGCAGAGAGCGA |
|  | Reserve: AGTGCAGGGTCCGAGGTATT |
| miR-613 | Forward: GCGCGAGGAATGTTCCTTC |
|  | Reserve: AGTGCAGGGTCCGAGGTATT |
| TSIX | Forward: AGGCCTTCGGTCCAATTCAG |
|  | Reserve: GCAGAAGGTGGAAGGCTCAT |
| LOC101926935 | Forward: GGTAGCACCAAGAAGGTCCC |
|  | Reserve: AGAGACTGGACCGAGAGCAT |
| KCNQ1OT1 | Forward: ACTCACTCACTCACTCACT |
|  | Reserve: CTGGCTCCTTCTATCACATT |
| circ_0020316 | Forward: CCAAGGCACACAGGGTACTT |
|  | Reserve: CTCATGCTCTGTCAGCCGAA |
| circ_0091807 | Forward: CCAAGGCACACAGGGTACTT |
|  | Reserve: CTCATGCTCTGTCAGCCGAA |
| circ_0091808 | Forward: AGTGCGATGTCGTTTTGTGC |
|  | Reserve: GGGTGGAAAGGAGCCACAAG |
| GAPDH | Forward: GAAGGTGAAGGTCGGAGTC |
|  | Reserve: GAAGATGGTGATGGGATTTC |
| U6 | Forward: CTCGCTTCG GCAGCACA |
|  | Reserve: AACGCTTCACGAATTTGCGT |

**Supplementary Table 2** The target binding scores of miRNAs to MAPK3

| miRNA | mirDIP (Integrated Score) | miRWALK (score) | DIANA TOOLS (miTG score) |
| --- | --- | --- | --- |
| miR-767-3p | 0.202623777 | -19.9 | 0.740595748 |
| miR-761 | 0.298625762 | -21.9 | 0.759272947 |
| miR-92a-2-5p | 0.348438656 | -23.1 | 0.955738758 |
| miR-4270 | 0.255721422 | -30.1 | 0.833594722 |
| miR-1321 | 0.230202353 | -16.7 | 0.847981425 |
| miR-483-5p | 0.344499987 | -20.4 | 0.745993258 |
| miR-423-5p | 0.36660002 | -26 | 0.830781544 |
| miR-613 | 0.622444284 | -22.8 | 0.86809789 |

**Supplementary Table 3** The binding of lncRNAs to miRNAs

| LncRNA | DIANA-LncBase (score) | | | |
| --- | --- | --- | --- | --- |
|  | miR-92a-2-5p | miR-4270 | miR-423-5p | miR-613 |
| TSIX | 0.755 | 0.914 | 0.836 | 0.883 |
| LOC101926935 | 0.975 | 0.870 | 0.938 | 0.794 |
| LL22NC03-86G7.1 | 0.978 | 0.997 | 0.963 | 0.798 |
| AC006548.28 | 0.998 | 1.000 | 0.976 | 0.996 |
| XLOC_011484 | 0.793 | 0.843 | 0.905 | 0.757 |
| RP1-309I22.2 | 0.926 | 0.995 | 0.808 | 0.932 |
| CTA-796E4.5 | 0.934 | 0.826 | 0.921 | 0.894 |
| LL22NC03-22D1.1 | 0.890 | 0.836 | 0.713 | 0.715 |
| CTA-292E10.9 | 0.870 | 0.969 | 0.757 | 0.703 |
| RP3-323A16.1 | 0.999 | 0.998 | 0.964 | 0.954 |
| KCNQ1OT1 | 1.000 | 1.000 | 0.995 | 0.990 |

**Supplementary Table 4** The binding of circRNAs to miRNAs

| CircRNA | CircBank (length) | | | |
| --- | --- | --- | --- | --- |
|  | miR-92a-2-5p | miR-4270 | miR-423-5p | miR-613 |
| circ_0020316 | 8519 8242 7722 | 2136 3008 3118 6096 8414 2655 | 4973 1903 | 4429 |
| circ_0091807 | 4131 4518 | 6572 2722 7061 929 | 3632 3426 5657 | 290 25 358 997 |
| circ_0091808 | 4852 5239 | 7293 3443 7782 1650 | 4353 4147 6378 | 1011 746 1079 1718 |
